# Primary healthcare nurses’ experiences in managing chronic diseases during COVID-19 in the North West province

**DOI:** 10.4102/phcfm.v16i1.4491

**Published:** 2024-09-30

**Authors:** Sheillah H. Mboweni

**Affiliations:** 1 Department of Health Studies, College of Health Sciences, School of Social Sciences, University of South Africa, Pretoria, South Africa

**Keywords:** COVID-19, experiences, management, patient with chronic disease, primary healthcare, professional nurses

## Abstract

**Background:**

The World Health Organization, stated that the coronavirus disease 2019 (COVID-19) pandemic not only affected the socioeconomic well-being of millions but also had adverse effects on public health, particularly in the management of chronic diseases at the primary healthcare (PHC) level. What remained unknown was the experiences of professional nurses(PNs) working in PHC regarding this issue.

**Aim:**

The study aimed to explore and describe the lived experiences of PHC nurses in managing chronic diseases during the COVID-19 pandemic.

**Setting:**

The study was conducted in the North West province, South Africa.

**Methods:**

A qualitative descriptive phenomenological design was employed to collect and analyse data. Face-to-face interviews were conducted and audio recorded with 16 PNs from five high-volume PHC facilities selected purposively.

**Results:**

The study’s findings reveal four themes: suboptimal care for patients with chronic disease, a lack of resources, mental health challenges experienced by PHC nurses, and stigma and discrimination from both family and community members.

**Conclusion:**

The neglect of PHC and its frontline healthcare staff has impeded the mental health of PHC workers and the management of chronic diseases thus any progress made in reducing the burden of chronic diseases is likely to have regressed during the COVID-19 pandemic.

**Contribution:**

Policymakers should prioritise strengthening PHC by implementing integrated disease management policies, ensuring ethical clinical standards, providing supportive supervision, fair resource allocation and capacity building for PHC staff. In addition, addressing stigma and discrimination, and raising awareness among families and communities is crucial for future pandemics to effectively manage both chronic and infectious diseases.

## Introduction

The coronavirus disease of 2019 (COVID-19) pandemic posed unprecedented challenges to healthcare systems worldwide, including the management of chronic diseases.^1^ Primary healthcare (PHC) nurses play a crucial role in providing comprehensive care to individuals with chronic conditions.^2^ However, the impact of the pandemic on these nurses and their experiences in managing chronic diseases in specific regions remains a topic of significant interest and concern. Chronic disease and non-communicable disease (NCD) services were cut back in several nations, including South Africa, because of the COVID-19 pandemic’s intense political polarisation.^3^ This strategy runs counter to the investment of the World Health Organization (WHO) in the prevention, control and management of chronic illnesses, as well as the African region’s White Paper on Healthcare System Transformation, which includes respect for human rights.^4^ During emergencies, there is a tendency to prioritise infectious disease outbreaks and overlook the management of chronic diseases, neglecting their significance as a crucial component of emergency response.^5^ Human immunodeficiency virus (HIV) has been recognised as a chronic disease, warranting attention and management like other long-term health conditions.^6^ According to the WHO,^6^ chronic diseases are defined as persistent conditions that progress gradually and this classification includes NCDs as well as infectious diseases such as HIV over the years, the treatment and care of HIV have undergone significant advancements, shifting it from a catastrophic outbreak to a controllable chronic illness. This transformation has been achieved through the lifelong utilisation of antiretroviral therapy (ART).^5,6^ Chronic diseases are the leading cause of illness and death on a global scale, resulting in the annual loss of 41 million lives. Among these fatalities, 15 million occur prematurely, affecting individuals between the ages of 30 and 70 years. It is worth noting that the premature mortality rate is higher among men, accounting for 16.4%, compared to women at 11.8%.^7^ Chronic diseases encompass a range of conditions, including hypertension, stroke, coronary heart disease heart failure, diabetes mellitus, cancer, chronic obstructive pulmonary disease and bronchial asthma, primarily affecting the cardiovascular and respiratory systems.^8^ The impact of chronic diseases in low- and middle-income countries is increasing significantly.^9^ In these regions, chronic diseases account for a substantial percentage of fatalities, with over 90% of deaths from chronic obstructive pulmonary disease, and over 80% from heart disease and diabetes mellitus.^10^ In adiition, in developing nations, these chronic diseases are responsible for approximately 85% of these deaths.^10,11^ Premature mortality rates are significant in these countries, fuelled by unhealthy lifestyles.^6^ Consequently, the WHO has established a goal to reduce premature deaths from NCDs by 25% by 2030, aligning this with the United Nations (UN) Sustainable Development Goal (SDG) 3.4.^6^ This objective aims to achieve prevention, treatment and promotion of mental health and well-being.^12^ The quadruple burden of disease initiative plays a significant role in addressing the SDGs and achieving universal health coverage by 2030. However, its progress was impeded by a shift in attention towards infectious diseases. In response, the WHO recommended the decentralisation of the chronic care model (CCM) to PHC settings and task shifting in low- to middle-income countries, particularly in sub-Saharan Africa. These measures aimed to address the limited resources, such as a shortage of healthcare professionals, by empowering PHC PNs to provide comprehensive care.^13^

The appearance of the COVID-19 pandemic further exacerbated the burden on an already overwhelmed healthcare system.^14^ This strain was particularly felt by PHC nurses.^14^ As of 2019, the prevailing chronic health condition in South Africa was hypertension, affecting a total of 4.74 million individuals.^15^ Furthermore, South Africa stands as the country with the largest HIV epidemic globally, with approximately 8.5 million people living with HIV (PLHIV). Comorbidity is prevalent among PLHIV in South Africa, with approximately 1.68 million individuals diagnosed with both HIV and a chronic health condition.^15,16^ Moreover, out of the country’s total population of 60.2 million, around 5.5 million PLHIVs are receiving ART.^16,17^ Integrated comprehensive management of chronic diseases during epidemics is important instead of cutting back access to chronic care services, as it reverses the progress made to achieve SDG 3 and quality of life through the implementation of the CCM.^18^ During the pandemic, there was a significant decline in emergency department appointments and in-person clinic visits for chronic conditions. This decrease had a widespread adverse effect on clinical decision-making worldwide, as it limited the ability to conduct physical examinations and laboratory tests.^19^ Moreover, patients faced reduced opportunities to receive community-based support and care for their chronic conditions.^18^ The global mortality rate associated with chronic diseases remains alarmingly high, and it is projected that three-quarters of all deaths worldwide will be attributed to NCDs by 2030.^6,11,18^ Therefore, the neglect of PHC during the pandemic is a worrisome matter.

The devastating neglect of PHC as a cornerstone of the health system resulted in 90% of the countries globally reporting a regression of health services, and South Africa was one of those regions.^20,21,22^ In South Africa, the primary quintuple burdens of chronic diseases are cardiovascular disease, HIV, cancer, diabetes and chronic respiratory diseases.^15^ Unfortunately, the country lacks a comprehensive and integrated plan to address the burden of these diseases during medical emergencies. The North West province, like many other regions, encountered specific challenges and adaptations in delivering healthcare amid the pandemic. Gaining insights into the encounters of PHC nurses within this context is crucial for identifying areas for improvement, addressing gaps in care and developing effective strategies to mitigate the impact of COVID-19 on chronic disease management. The study was conducted in a high-burden district for HIV and AIDS, tuberculosis and NCDs, primarily in a rural area with a significant population of migrant workers engaged in farming, mining and tourism activities. This region is densely populated. According to Ajaero et al., the prevalence of NCDs is higher among migrants (19.81%) compared to non-migrants (16.69%) because of inadequate access to health services.^23^ In addition, there is inadequate literature on the experiences of professional nurses (PNs) in PHC settings, as most studies have been conducted in hospital settings where patients with COVID-19 were admitted. This project aimed to fill this gap by exploring the lived experiences of PHC nurses in the North West province of South Africa in managing chronic diseases during the COVID-19 pandemic. The objective was to provide evidence-based interventions and recommendations to policymakers regarding the integration of the management of chronic diseases during pandemics and disasters.

## Research methods and design

### Study design

A qualitative descriptive phenomenological approach was employed. The primary aim of employing descriptive phenomenology in this study was to comprehensively capture and describe the fundamental nature and framework of the phenomenon being studied, which in this case was the experiences of PHC PNs as they navigated the management of patients with chronic illnesses amid the COVID-19 pandemic.^24,25,26^ Through this approach, the researcher acquired a detailed and comprehensive understanding of the nurses’ personal experiences and perspectives and the obstacles they encountered.

### Study setting

The study was conducted in the Bojanala district located within the North West province of South Africa, specifically in five high-volume public PHC facilities. The district is primarily rural and has a substantial population of migrant workers. It is characterised by high population density, informal settlements, farming and mining areas. The district serves as an economic hub for the province and experiences a significant occurrence of chronic diseases, HIV/AIDS and tuberculosis. This prevalence is attributed to various socioeconomic factors, including poverty, a large population of migrant labourers, overcrowding, substandard housing conditions, limited access to healthcare services, unemployment and a lack of education. Moreover, unhealthy lifestyle behaviours contribute to the weakening of the immune system, rendering individuals more susceptible to chronic diseases, TB, HIV and AIDS infections.^27^

### Population and sampling strategy

The participants of the study were PHC nurses who were actively engaged in the direct care and management of chronic diseases throughout the COVID-19 pandemic, with the aim of gathering and exploring their lived experiences. A purposive sampling technique was employed to select 16 PNs who voluntarily expressed their willingness to contribute and share their experiences. The initial participants were purposefully selected, as nurses directly managing chronic diseases during the COVID-19 pandemic and subsequent participants were recruited through consecutive sampling where the researcher and participants recruited other PNs who were available that day and willing to participate in the study as this target group was relatively small. All participants fulfilled the inclusion criteria of being actively employed in PHC facilities during the COVID-19 pandemic in 2020 and beyond.^25,26^

### Data collection

Semi-structured individual face-to-face interviews were conducted to determine the PNs’ experiences regarding the management of chronic diseases during the COVID-19 pandemic. Facilities were approached after obtaining permission to conduct the study from the North West Provincial Department of Health and the Bojanala District management team. The facility managers played a crucial role in facilitating access to PHC PNs who were willing to participate in the study. Consent forms and information leaflets were distributed via email, ensuring that all PNs involved in managing chronic diseases received the necessary documentation. Appointments were then scheduled with the PNs who expressed their willingness to participate, allowing for data collection to take place.

A preliminary study was first performed in a facility that was excluded from the main study. Data collected and analysed from this pilot study were not incorporated into this study, However, it provided an opportunity for the researcher to enhance the research questions and improve the interviewing skills. Participants provided informed written consent, granting permission for the audio recording of the interviews. The interview sessions were held in a private room, without interruptions allowing participants to articulate their perspectives, emotions and personal experiences. These took place during participants’ tea and lunch breaks to avoid interruption of service delivery. The district and the university’s protocols and regulations regarding infection prevention and control during the COVID-19 pandemic were carefully followed. This included ensuring proper ventilation in the interview room, maintaining a distance of 1.5 m between individuals, and providing hand sanitisers and masks for the participants. Participants were asked the following question: ‘What has been your personal experience of managing chronic diseases during the COVID-19 pandemic?’ Probing questions were utilised for clarification if participants required further understanding. The interviews were conducted in both English and Setswana, the local language, to facilitate comprehension and elicitation of responses. The data collected during the interviews were later translated into English during the transcription process. The researcher, having worked in the province for 8 years, possesses fluency in Setswana and can comprehend and communicate in the language. The interviews lasted from 45 min to an hour. Field notes were taken to capture non-verbal cues and participants’ emotional responses. The data collection process took place from April to June 2022. Data saturation was achieved with 16 participants, and data saturation was reached when additional participants no longer provided new information and when emerging themes became repetitive. This saturation point can typically be achieved within a range of 12 to 25 participants.^28^

### Data analysis

Data analysis was conducted simultaneously with the data collection process. The descriptive phenomenological analysis approach, consisting of four steps (bracketing, intuiting, analysing and describing), was employed to examine the lived experiences of PNs.^24,25,26^ During the bracketing phase, the researcher impartially suspended their personal perspectives and viewpoints regarding COVID-19 and the management of chronic disease patients. A reflexive journal was maintained by identifying possible areas of pre-existing knowledge or situations that could lead to conflicts in roles, the researcher made note of intriguing observations. The involvement of gatekeepers helped ensure neutrality. The data were transcribed word for word without alterations, and the researchers extensively reviewed and familiarised themselves with the transcriptions, organising similar data into distinct categories and themes. During the intuiting phase, the researcher maintained an open mindset to explore various interpretations by discussing the transcribed data and emerging themes with a seasoned researcher, ensuring the integrity and quality of the analysis. In the analysis phase, noteworthy statements were extracted, data were organised into categories and the fundamental meaning of the phenomenon being studied was understood. Finally, in the descriptive phase, the researcher reached a comprehensive insight into the experiences of PNs and described their challenges.^24,25,26^

### Ethical considerations

The study adhered to the principles outlined in the Nuremberg Code, which governs the ethical treatment of human participants in research activities. Ethical clearance to conduct this study was obtained from the University of South Africa, College of Human Sciences Research Ethics Committee (No. 90476050_CREC_CHS_2021). The study received approval from the North West Provincial Department of Health and the Bojanala District Institution Review Board before commencing. Written consent, voluntarily given by the participants, was obtained. To ensure confidentiality, numerical identifiers were used instead of the primary nurses’ names. To adhere to ethical principles and comply with the Protection of Personal Information (POPI) Act, which became effective in April 2020 in South Africa, no personally identifiable information of the institution or the participants was included in the study.

The audio recordings were securely stored in a locked location, separate from the consent forms. Field notes and verbatim data were electronically saved and safeguarded with password protection. Access to this data is restricted solely to the researcher to guarantee anonymity, confidentiality and data security. Participants were explicitly informed of their prerogative to withdraw from the study at any stage without incurring any adverse consequences.

### Trustworthiness in qualitative research

Trustworthiness is a way of ensuring data quality or rigour in qualitative research.^26^ The trustworthiness of the whole study was guaranteed by using the model of Lincoln and Guba,^28^ comprising a set of criteria, the study aimed to ensure the credibility, dependability, confirmability and transferability of the research findings. To improve the trustworthiness of the study, several strategies were employed. These included utilising a phenomenological design, employing purposive sampling methods and conducting recorded interviews to capture participants’ lived experiences, to gain a deeper understanding and insight into the phenomenon being investigated.^24^ Reflexibility refers ‘to the researcher’s self-awareness and was used to guard against personal bias in making judgements and to enhance the quality of the study’.^24,25,26^ Dependability was reinforced by implementing measures to enhance credibility, such as dedicating additional time to the research process by spending about 45 min to 01:00 with participants or more depending on the participant’s response. To ensure the accuracy and reliability of the findings, a member check was conducted. This involved the researcher revisiting participants to validate if their experiences had been accurately captured and represented in the study.^25^ Furthermore, to enhance transferability, comprehensive information on the lived experiences of PNs from various facilities within the district was collected until data saturation was achieved including a pilot study. Confirmability was strengthened by maintaining audit trails of the audio-recorded interviews and meticulously documenting field notes, which included the study’s findings. The data analysis and themes generated from the study were shared with an expert in qualitative research for a thorough examination of the process. Feedback and discussion ensued, aimed at strengthening the rigour and credibility of the study while minimising potential biases.^26,27,28^

## Results

### Characteristics of participants

The gender distribution indicates a higher representation of female participants than males, with approximately two-thirds of the participants being female as summarised in Table 1. This gender imbalance may reflect the overall gender composition of the PHC PN workforce or the willingness of female nurses to participate in the study. In terms of work experience, most participants fell into the 6–10 years and 11–15 years categories, comprising around two-thirds of the total participants. This suggests that the study included a relatively experienced group of PHC PNs. It is worth noting that there were fewer participants with higher levels of work experience (21–25 years and 25 years and above), which might be because of factors such as retirement or a smaller pool of nurses with extensive experience in the selected facilities. These participant characteristics provide insight into the composition of the sample and highlight the representation of different genders and levels of work experience among the PHC PNs involved in the study.

**TABLE 1:** Participants’ characteristics.

No.	Code	Gender	Working experience in years
1	PN 1	F	5
2	PN 2	F	7
3	PN 3	F	8
4	PN 4	F	9
5	PN 5	F	5
6	PN 6	F	11
7	PN 7	M	23
8	PN 8	M	22
9	PN 9	F	30
10	PN 10	F	11
11	PN 11	F	12
12	PN 12	F	9
13	PN 13	M	7
14	PN 14	M	6
15	PN 15	F	11
16	PN 16	M	11

PN, Participant number.

### Themes and subthemes

The study findings reveal four main themes and their corresponding subthemes, which offer a comprehensive understanding of PHC PNs’ experiences in managing chronic diseases during the COVID-19 pandemic. These themes and subthemes are summarised in Table 2 for a concise representation of the study’s key findings.

**TABLE 2:** Themes and subthemes.

Themes		Subthemes
1.	Suboptimal care of patients with chronic diseases during the COVID-19 pandemic	Strict adherence to COVID-19 regulations rather than comprehensive care Challenges to priority programme performance A high number of patients disengaged in care and policy changes Focus on the prevention and medical management of COVID-19 patients. than patients with chronic diseases A lack of information regarding the management of COVID-19 at PHC level
2.	Shortage of resources affected the management of chronic diseases	Shortage of material resources Infrastructural challenges Shortage of human resources
3.	Mental health challenges experienced by PHC PNs in managing chronic diseases	Stress and depression A lack of psychosocial support Inadequate supportive supervision
4.	Stigma and discrimination suffered by PHC PNs managing chronic diseases	From family members From the community

COVID-19, coronavirus disease 2019; PHC, primary healthcare; PN, professional nurse.

#### Theme 1: Suboptimal chronic care of patients with chronic diseases during the COVID-19 pandemic

The theme of suboptimal chronic care encompasses several subthemes. Participants revealed that there was greater emphasis on strict adherence to COVID-19 regulations rather than providing comprehensive care. A significant number of patients disengaged from their medical obligations, posing challenges to effective care delivery. Efforts were directed towards prevention and medical management specifically tailored to COVID-19 patients. More emphasis was on the management and interventions for this population while neglecting chronic diseases and other health conditions.

**Strict adherence to COVID-19 regulations rather than comprehensive care:** Participants expressed concerns about the impact of strict adherence to COVID-19 regulations on comprehensive care, affecting the ethical standards of clinical care for patients with chronic diseases. The pandemic posed an ethical dilemma for PHC nurses, as they had to choose between prioritising their self-protection or fulfilling their expected role in providing clinical care, jeopardising their professional ethics and moral values (Ubuntu). Furthermore, the COVID-19 pandemic resulted in the neglect of newly emerging chronic diseases and TB cases because of overlapping symptoms with COVID-19 and other chronic illnesses. This lack of testing led to delays in treatment, increasing the risk of infection transmission to family members and complications arising from undiagnosed and untreated chronic diseases. Participants highlighted this with concern:

‘We encountered challenges in identifying TB cases, as we initially attributed the symptoms to COVID-19. As a result, TB testing was not conducted until later when we decided to test for both TB and COVID-19.’ (P1, F, 5 years)

‘The priority shifted from putting the patients first to prioritizing the safety of healthcare workers. Our professional, ethical, and moral principles were overshadowed by fear.’ (P8, M, 22 years)

Ubuntu is an African philosophy that describes a set of African-origin value systems that emphasise humanity, compassion, respect, empathy, kindness, caring, generosity and connectedness.

Patients expressed dissatisfaction with the quality of chronic care services delivered by PHC nurses, as essential aspects of care such as physical examinations, adherence counselling, vital sign monitoring and necessary tests were neglected during regular clinical reviews. This dissatisfaction was evident through both verbal and nonverbal expressions of the patients. The nurses had to prioritise their COVID-19-related responsibilities, leading to diverted attention away from providing comprehensive care for patients with chronic diseases. Participants stated with sadness the following:

‘We didn’t even perform physical examinations. Patients feel dehumanized and neglected as if we don’t care about them. It appears that we merely dispense treatment without genuine concern.’ (P7, M, 23 years)

‘Insufficient education and adherence counselling were provided, which could have helped reduce the time of exposure.’ (P1, F, 5 years)

**Challenges to priority programme performance:** Participants disclosed that the pandemic led to the disruption of essential services and programmes as healthcare systems shifted their focus and resources towards managing COVID-19 cases. This diversion of attention and resources impacted the delivery and performance of other priority programmes, such as chronic diseases, maternal and child health, HIV, AIDS and TB:

‘We were so consumed with COVID-19 that other important screenings and tests took a backseat.’ (P2, F, 7 years)

‘Child health services, HIV and TB services suffered immensely as our attention was diverted towards managing the pandemic, we were also not managing child health well, we immunise without weighing the child, [*s*]hortage of vaccine was also a problem.’ (P15, F, 11 years)

Participants disclosed that the COVID-19 pandemic strained the financial and human resources allocated to priority programmes. Increased demands led to redirected budgets towards pandemic response, diminishing funding for other programmes and compromising their effectiveness. Lockdowns, travel restrictions and disruptions in supply chains further hindered the availability of essential commodities, medications and equipment necessary for these programmes. As a result, access to and utilisation of health services were reduced, routine data collection and monitoring systems were disrupted, and delays, shortages and challenges in maintaining programme performance arose. The participants stated the following sentiments:

‘The neglect of priority programs has had a significant impact on chronic disease management, there was no integrated screening tool, policies or protocol for both COVID-19, other infectious and chronic diseases, TB and HIV and nutritional status leaving many patients unattended and at risk and TB testing overlooked.’ (P4, F, 9 years)

Participants stressed that the neglect of fundamental aspects of outreach programmes such as screening and testing hindered progress, leading to poor performance in various PHC indicators such as screening and diagnoses of new patients with chronic diseases, TB, HIV, including immunisation:

‘The community outreach program has experienced notable disruptions, with Community Health Workers (CHWs) and Outreach Team Leaders (OTLs) initially being halted and later resuming their activities. However, their focus has shifted primarily towards COVID-19 screening. Many of them were above the age of 60, have comorbidities, and are at a higher risk for COVID-19.’ (P13, M, 7 years)

**A high number of patients disengaging in care and policy changes:** Primary healthcare nurses observed a significant rate of patient disengagement in chronic disease care, including HIV, with many patients relocating to their home province or country because of employment and COVID-19 restrictions. Patients consistently demonstrated non-compliance by neglecting treatment plans, missing appointments, disregarding important clinical review dates, failing to provide specimens for testing (e.g. blood for viral load testing) and neglecting the use of condoms. Participants highlighted that:

‘We experienced a significant loss in follow-up rates as many patients returned to their home provinces such as Gauteng, Northern Cape, and Eastern Cape, or even outside South Africa to countries like Lesotho, Mozambique, Zimbabwe, and Botswana. These individuals initially came to our area for work in the mines and had to wait until the lockdown regulations were lifted. Monitoring tests were not done at all.’ (P6, F, 11 years)

Participants revealed that policy changes were made in response to COVID-19 concerns and restrictions, affecting the collection of medications for chronically stable patients. To minimise the risk of infection and reduce overcrowding, modifications were made to the Central Chronic Medicines Dispensing and Distribution (CCMDD) programme. Newly diagnosed patients were included, and the criteria for considering patients stable on treatment was reduced from 12 to 6 months. These changes had implications for monitoring patients’ response to chronic treatment and overall programme monitoring. Participants reported the following:

‘To ensure continuity of care, we provided newly diagnosed patients with a three-month treatment supply and review, even during the early stages of their treatment.’ (P10, F, 11 years)

‘The criteria for a 12-month stable treatment period was revised to six months, accompanied by three-month medication supplies. This adjustment aimed to manage overcrowding and reduce long waiting hours.’ (P3, F, 8 years)

Participants reported a significant decrease in clinic attendance, referred to as headcount, as patients with chronic diseases and others were hesitant to visit clinics because of fear. This decline in patient influx negatively affected the overall performance of healthcare facilities. To tackle this issue, tracers were suggested to locate and contact these patients through telephone communication. Community health workers (CHWs) also visited households and collaborated with non-government organisations to assist patients in returning to care. Although some patients remain lost to follow-up, the situation is gradually improving and returning to normal. The participants emphasised the following points:

‘Due to fear and concerns regarding COVID-19, a significant number of patients were reluctant to visit the clinic, resulting in a high rate of missed appointments and this requires employment of more facility telephone tracers that the department does not have and rely on Implementing partners/NGOs except CHWs who conduct household visit[*s*].’ (P11, F, 12 years)

**Focus on the prevention and medical management of COVID-19 patients rather than chronic disease management:** The PHC nurses highlighted that the primary focus was on preventing and medically managing COVID-19 patients, overlooking the importance of addressing chronic conditions, and health promotion programmes. This neglect of fundamental aspects such as screening and testing hindered progress, leading to poor performance in various PHC indicators. Participants highlighted with concern that:

‘I am currently employed in the outpatient department, and our focus has primarily been on COVID-19, with limited attention given to screening and testing for chronic and other infectious diseases.’ (P2, F, 7 years)

‘Patients who tested positive for COVID-19 were given expedited care and prioritized over others.’ (P9, F, 30 years)

**A lack of information related to COVID-19 management at primary healthcare level:** Participants felt that while they were previously prioritised for improving their management of chronic diseases, they were not given the same priority for COVID-19 training. As a result, they lacked the necessary knowledge and skills to handle patients with both chronic diseases and COVID-19 (comorbidity) at the PHC level. In addition, participants faced a lack of access to information and communication technology (ICT) resources, which hindered their ability to attend online training mandated by their healthcare facilities. Consequently, they had a limited understanding of COVID-19, despite being the first point of contact for community and patient care:

‘We possess the essential knowledge, skills and confidence to effectively manage chronic diseases, minor ailments, and infectious diseases. Our training includes guidelines, except for COVID-19, we were told to attend online, and network in our clinic is a problem and I have to use my own phone, no laptop.’ (P14, M, 6 years)

#### Theme 2: Shortage of resources affected the management of chronic diseases

**Shortage of material resources:** Participants reported that they encountered difficult work environments marked by a lack of necessary protective gear, medications, supplies, and essential tools like blood pressure and glucose monitoring devices. As a result, this situation contributed to substandard care for patients with chronic illnesses and other health issues. Participants highlighted the following:

‘We had no gloves, mask[*s*], [*or*] gown[*s*] and sometimes we repeat it for 3 days or a week. Priority was hospitals.’ (P15, F, 11 years)

‘Some chronic drugs were out of stock as we were told to give patients three monthly supply.’ (P9, F, 30 years)

Participants emphasised that the shortage of PPE was not limited to the clinic alone. They pointed out that even the emergency response services (EMRS) team, responsible for transferring patients with complications related to chronic diseases, and suspected or confirmed COVID-19 infections to the hospital, faced a scarcity of PPE. As a consequence, the delayed access to specialised emergency care for chronic diseases led to instances where individuals experienced strokes:

‘EMRS also did not have PPE, when you call the ambulance, EMRS staff was also afraid of COVID-19 infection once you said it is a suspected or confirmed COVID-19 case, they will be hesitant to come and get the patient.’ (P9, F, 30 years)

**Infrastructural challenges:** Participants mentioned that they worked in an environment with poor infrastructure, which included working in congested or poorly ventilated facilities. These conditions further exacerbated their mental health issues and poor management of chronic diseases. Participants brought to attention the following:

‘My clinic is very small with a small window and not enough space to practice physical distancing, ventilation was poor.’ (P8, M, 22 years)

**Shortage of human resources:** Participants reported that most healthcare workers were redirected or reassigned to COVID-19 response efforts, in hospitals leading to staff shortages in PHC and reduced capacity for delivering priority programmes, and chronic disease management. In addition, healthcare workers themselves faced increased stress, fatigue and burnout, impacting their ability to perform their duties of managing chronic diseases effectively:

‘God protected us, we were depressed, and we had a serious shortage of staff, imagine having 10/20 staff being positive or in quarantine.’ (P11, F, 12 years)

#### Theme 3: Mental health challenges experienced by primary healthcare professional nurses managing chronic diseases

Professional nurses suffered prolonged stress that led to depression because of a lack of supportive supervision, psychosocial support, an employee assistance programme for counselling, and debriefing.

**A lack of psychosocial support:** Participants emphasised the insufficient psychosocial support available during the challenging period of the pandemic, especially for individuals who were diagnosed with COVID-19 and required isolation or quarantine. They highlighted the absence of assistance in addressing their emotional and psychological needs during this difficult time. This situation negatively impacted PHC nurses’ ability to provide effective chronic disease management, education and adherence counselling to patients. Participants conveyed their emotions with tears streaming down their faces:

‘I was in isolation, full of fear of losing my life, not a single manager called or came to see me, except the national tracing call even after coming back, I continued working as if nothing happened.’ (P16, M, 11 years)

Participant disclosed that they did not receive counselling or debriefing support following the pandemic, even after and they perceived the study interview as a chance to articulate their personal struggles, challenges and feelings of grief:

‘Even, now we never had counselling, I just feel like talking to you is a debriefing or counselling session.’ (P10, F, 11 years)

‘I have never been stressed like that in my entire nursing career, we never saw department social workers or psychologists coming to work, just to support us but nurses were [*on-*] duty daily.’ (P7, M, 23 years)

**A lack of supportive supervision:** Participants expressed disappointment and emotional distress because of the lack of supportive supervision from their managers or supervisors. They tearfully conveyed their perception that nurses and healthcare providers at the hospital level received more support and attention compared to PHC nurses. These PHC nurses serve as the initial point of contact for all community members and work tirelessly to reduce the burden of chronic diseases in their communities. Participants reported the following:

‘Not a single PHC or provincial manager came to visit our CHC during the COVID-19 pandemic.’ (P5, F, 5 years)

**Stress and depression:** Participants with no or poor coping mechanisms suffered high levels of stress that ended up in depression. This was made worse by isolation from their family members. The shortage of resources, poor infrastructure and neglect of PHC nurses exacerbated the situation. This affected PNs to provide quality chronic disease management:

‘We worked under high levels of stress, I was so depressed at the time but continued working, we had no option, we needed a salary to take care of our families.’ (P2, F, 7 years)

‘I was stressed and afraid daily when consulting patients whether positive or not.’ (P4, F, 9 years)

#### Theme 4: Stigma and discrimination suffered by primary healthcare professional nurses managing chronic diseases

Participants reported the stigma and discrimination that they faced as PHC nurses from their family members, including spouses, as well as community members that they had served for so many years in various public settings, such as taxis or buses, and shopping complexes. They had no one to share what they experienced at work. This discrimination stemmed from the fear of contracting COVID-19, with the assumption that these nurses were at high risk of exposure to the virus.

**Stigma and discrimination from family members:** Participants shared that the support from their families was significantly impacted during the COVID-19 pandemic. Primary healthcare nurses expressed feeling isolated and separated from their family members, as they had to stay in their rooms to avoid the risk of contracting the virus. These experiences were described emotionally by the participants, with tears in their eyes:

‘My partner told me to use the outside room so that I don’t infect him and the kids.’ (P5, F, 5 years)

‘Everyone at home including my children will go to their rooms when I come back from work.’ (P8, M, 22 years)

**Community stigma and discrimination:** Primary healthcare nurses previously enjoyed a high level of community respect, but they now face concerns about stigmatisation and discrimination because of fears of being carriers of COVID-19. People refuse to share transportation and taxi drivers ignore their requests for rides, making it challenging for nurses to commute to work. This fear of COVID-19 threatens the principle of Ubuntu, which emphasises community interconnectedness and support. Consequently, nurses feel the need to change out of their uniforms to conceal their professional identity:

‘I had no car; people get out of the taxi when we come in uniform.’ (P5, F, 5 years)

‘Taxi driver just drove past me as if he did not see that I’m stopping him, it was raining and getting late.’ (P9, F, 30 years)

## Discussion

The four key themes that emerged from PHC nurses’ experiences in managing chronic diseases during the COVID-19 pandemic in the Northwest province are discussed. These themes provide insights into the healthcare system’s response to the pandemic and its impact on chronic disease management. This helps identify areas that need attention and improvement to ensure the provision of high-quality chronic care services.

### Suboptimal chronic care of patients with chronic diseases during the COVID-19 pandemic

The study highlights the inadequate focus on chronic care in PHC settings, resulting in insufficient care for patients with chronic diseases. Objective clinical assessments, physical examinations and necessary tests were neglected because of strict adherence to COVID-19 regulations and limited knowledge about the virus. This disregard for essential healthcare practices contradicts nursing ethics and Ubuntu principles.

Nurses face ethical dilemmas regarding the choice between protecting themselves or providing care to the patients.^29^ This notion was supported by a study conducted in Iran and revealed that PHC nurses encountered ethical dilemmas where they were faced with difficult decisions regarding prioritising their own well-being or that of the patients under their care.^30^ Some nurses willingly endured hardships and ethical dilemmas out of their dedication to human health, reflecting the concept of Ubuntu in an African context.^30^ A systematic literature review reveals that nurses experienced mixed emotions, but some were able to overcome them through their professional dedication and compassion towards suffering and vulnerable patients, despite experiencing anxiety.^31^ Other studies indicate that nurses encountered ethical conflicts related to balancing self-care with comprehensive patient care, prioritising life and addressing intra- and inter-professional inequalities and resource limitations.^32,33^ These challenges affected the management of chronic diseases at the PHC level. The results of a study conducted in South Africa, involving patients with chronic diseases, confirmed these findings by revealing that they experienced inadequate clinical services from both nurses and the healthcare system as a whole amid the COVID-19 pandemic.^34^

The stringent adherence to COVID-19 regulations, which primarily focussed on the prevention and management of COVID-19 patients at the hospital level, rather than adopting a comprehensive integrated care approach for patients with chronic diseases and other conditions, had a devastating impact on the progress achieved in reducing the burden of diseases and implementing the CCM in PHC settings. The PHC, which is considered the foundation of the healthcare system, was disregarded during the response to the COVID-19 pandemic.^22^ This neglect led to setbacks in the prevention, control and management of chronic diseases, as well as other essential programmes.^22^ Some studies discovered that the COVID-19 pandemic not only resulted in suboptimal care but also brought a significant shift in the importance of sociocultural and religious practices in cases where treatment is absent for the diseases.^3^

Challenges such as loss to follow-up and disengagement in care and treatment were observed in this study and worsened by the provision of suboptimal care and strict adherence to COVID-19 regulations. This finding is consistent with a study conducted in South Africa, where disengagement in care was attributed to fear and uncertainty, worsened by COVID-19 vaccine hesitancy among individuals with chronic diseases.^35^ In contrast, a study conducted in Iraq reveals that patients in rural PHC settings did not believe that the virus existed, resulting in a large influx of patients to facilities.^36^

It is evident in this study’s findings that prioritisation of prevention and management of COVID-19 affected facility headcount, priority programmes aimed at reducing the burden of diseases such as TB, HIV and AIDS prevention, testing and treatment, management and control, maternal and child health especially immunisation, mental health and Gender based Violence (GBV) counselling services, Universal Health Coverage and NCDs screening and management. These cannot be under-prioritised and cannot be treated in silos. Another study conducted in South Africa affirmed low utilisation of PHC facilities and termination of mobile services because of fear of contracting the virus in the community.^34^ Confronting the global burden of diseases requires integrated efforts as they are closely interwoven including strengthening psychosocial support interventions in LMICs.^37^

Patients were introduced to the CCMDD programme early at 6 months after starting chronic medications instead of being stable in treatment for 12 months. Policy changes were introduced to enhance the continued engagement of patients with chronic diseases in care through repeat treatment prescription of treatment. An additional study conducted in South Africa affirmed that the implementation of the CCMDD system expanded access to chronic medication through community pick-up points, reducing overcrowding in healthcare facilities.^38^ The study further revealed that 88% of South Africa’s districts were registered with CCMDD, benefiting over 2 million individuals. Among them, 64% collected ART, 12% received ART and treatment for NCDs, and 24% managed chronic diseases only. While CCMDD strengthened the national differentiated service delivery (DSD) model, it posed challenges for newly diagnosed patients and those in their first 12 months of treatment, as clinical reviews and treatment monitoring were affected. These patients still needed close monitoring before receiving a 3-monthly treatment supply. An evaluation of the CMDD programme in South Africa revealed challenges for patients with chronic diseases, such as limited knowledge and refill access difficulties because of organisational issues.^39^ These challenges adversely affected treatment adherence. However, patients maintained a positive attitude because of the programme’s ability to reduce waiting times at public health facilities.^39^

Primary healthcare nurses, representing the healthcare system’s frontline, were neglected in COVID-19 training and workshops. This resulted in a lack of capacity building and information for PHC nurses regarding the virus, as the focus was on managing chronic diseases. This left nurses frustrated and uncertain about communicating with patients and the community they serve. A study conducted in Jordan supported the findings that nurses lacked knowledge of protocols and guidelines for the integrated management of COVID-19 and other conditions. This lack of knowledge contributed to psychological challenges and difficulties in effectively combating the virus.^39,40^ In South Africa, inadequate ICT infrastructure, insufficient support and limited ICT skills are prevalent, particularly in rural areas. This situation has a significant impact on online capacity-building efforts.^41,42^ Another study provided further support for the necessity of shifting from traditional medicine to telemedicine as a means to enhance access to chronic care.^43^ Patients with chronic diseases expressed the importance of utilising telemedicine, including telephone counselling, follow-up care, short message service reminders and a buddy system for medication refill collection.^43^ These measures aim to enhance access to care and reduce health disparities. This highlights the need for government advocacy to address educational and health inequalities in rural areas.

### Shortage of resources affected the management of chronic diseases

This study clearly shows that PHC faces a shortage of material and human resources. Hospitals were prioritised, leading to the reassignment of PHC staff to other roles worsened by the absence of staff because of illness, isolation or quarantine. As a result, the management of chronic diseases at the primary level of care was adversely affected. A study in Brazil demonstrates that the management of COVID-19 had both strengths and weaknesses, as a significant number of frontline health care workers (HCWs) were shifted to prevention and response activities, leading to a shortage of human resources and the neglect of priority programmes and the overall health system.^44^ Nurses’ reassignment to unfamiliar work environments severely affected nursing care and the nurse-patient relationship. Limited knowledge and skills in managing patients with COVID-19 further exacerbated the situation.^45^ Primary healthcare nurses were used to manage chronic diseases and not patients admitted to the hospital.^45^ A study in the United States of America (USA) found that critical staffing shortages in the healthcare system led to inadequate operational capacity and staff-patient ratios.^46^ To address this issue, capacity building for all nurses, including those in PHC, is crucial. This ensures that nurses are well-equipped to handle their responsibilities, even when reassigned to other duties.

Nurses face shortages of supplies, chronic medication and essential equipment like BP and glucose measuring machines, which hinder their ability to monitor patient conditions and may lead to delayed medical attention. Untreated chronic disease patients may face challenges with adherence, leading to complications, drug resistance and a negative impact on their health-related quality of life.^46,47,48,49^

### Mental health challenges experienced by primary healthcare professional nurses managing chronic diseases

It is evident in this study that PHC nurses experienced significant stress, despite not directly treating COVID-19 patients in their facilities. These factors contributed to PHC nurses experiencing stress, depression, anxiety, insomnia, moral injury, burnout, post-traumatic stress, and a lack of coping mechanisms and resilience.^48,50,51,52^ Furthermore, a systematic review and meta-analysis studies demonstrated that healthcare workers in general experienced high levels of stress, depression and insomnia.^53,54^

It is evident in study findings that the support provided by district health system managers was also a concern, and this is in line with a multi-survey study conducted in the USA, which revealed leadership failures that fractured worker relationships.^55,56,57^ Positive work dynamics, a supportive care context, adequate tools for care, team spirit, gratitude and family support were identified as key factors in helping nurses cope with challenges during the COVID-19 pandemic.^43,47,53^ Several studies revealed that factors such as a high number of deaths, inadequate preparedness, insufficient equipment and information, mental exhaustion and breakdowns resulted in burnout among nurses. Therefore, it is crucial to have measures in place during emergencies to mitigate burnout to enhance quality care.^58,59,60,61,62,63^

### Stigma and discrimination suffered by primary healthcare professional nurses managing chronic diseases

The study’s findings indicate that PHC nurses faced stigma and discrimination from their families and community members in the quest to manage chronic diseases. This finding is in line with studies conducted in Indonesia, China and the USA, which emphasised that stigma and discrimination negatively affected the relationship between nurses and patients.^46,64,65,66^ Addressing this issue is crucial. Nurses experienced social and societal stigma, even from their colleagues, highlighting a lack of knowledge on COVID-19 and the need for awareness.^40^ Similarly, studies conducted in Zimbabwe and Europe revealed that female frontline healthcare workers faced a higher risk of stigma and discrimination when infected, which had an impact on their mental health.^61,67^ This is significant as women constitute a larger proportion of the health workforce managing chronic diseases. On the contrary, a study conducted in Canada found that male nurses did not express significant struggles with the emotional and interpersonal aspects of their work and were able to support others.^62^ However, some male nurses reported using alcohol as a coping mechanism during the pandemic.^68^ Another study among Asian healthcare workers indicated that nurses were treated as ‘dirty’ and experienced discrimination from family members, highlighting the need for strong and integrated psychosocial support systems in healthcare.^55^ Furthermore, in a study conducted in India, nurses were stigmatised as potential sources of diseases, including certain social groups, races or classes, emphasising the importance of community education and awareness.^69^

### Strengths and limitations of the study

The research provides valuable insights into the neglect of PHC in managing chronic diseases during the COVID-19 pandemic, based on the experiences of PHC nurses in a high-burden district of the Northwest province. While the findings may not be universally applicable to the whole province, they serve as a basis for policymakers to develop integrated policies, interventions and protocols for future outbreaks.

## Recommendations

To effectively manage chronic diseases during a pandemic, policymakers must prioritise PHC as the foundation of the healthcare system and PHC nurses as the first contact of care in the community. This involves focussing on capacity building for PHC healthcare workers, improving ICT infrastructure for telemedicine, and facilitating training and communication. Integrated guidelines and protocols should be developed in collaboration with public health professionals to address the simultaneous management of the pandemic and chronic diseases. Policymakers should support collaborative awareness programmes, combat stigma and discrimination and prioritise psychosocial support for PHC workers. Adequate staffing, access to mental health services and peer support groups are essential. Evaluation of resource requirements, strategic resource distribution, and strong pharmaceutical partnerships and supply chain management systems are crucial. Ensuring timely availability of PPE, chronic medications and communication systems is essential.^44,70,71,72,73,74^

## Conclusion

This study sheds light on the experiences of PHC nurses managing chronic diseases during outbreaks. Findings reveal that PHC nurses face neglected capacity building for managing both chronic diseases and outbreaks, resource allocation challenges, fear, ethical dilemmas and compromised well-being. Staff shortages and a lack of support worsen the situation. These factors negatively impact patient outcomes, hinder the progress of priority programmes and compromise standards of care. Policymakers should integrate chronic disease management into pandemic responses, prioritise nurses’ mental health and provide support services. Further research and collaboration are needed to address challenges and promote well-being in future epidemics.
